# Pharmacokinetics of a Single Subcutaneous Extended‐Release Buprenorphine (Ethiqa XR) in Healthy Beagles

**DOI:** 10.1111/jvp.70082

**Published:** 2026-05-20

**Authors:** Kornelia Tiffinger, Hiroko Enomoto, Ronald E. Baynes, Laura M. Neumann, B. Duncan X. Lascelles, Masataka Enomoto

**Affiliations:** ^1^ Translational Research in Pain Program, College of Veterinary Medicine North Carolina State University Raleigh North Carolina USA; ^2^ Department of Population Health and Pathobiology, College of Veterinary Medicine North Carolina State University Raleigh North Carolina USA; ^3^ Department of Anesthesiology, Center for Translational Pain Research Duke University Durham North Carolina USA; ^4^ Thurston Arthritis Center University of North Carolina Chapel Hill North Carolina USA; ^5^ Comparative Pain Research and Education Centre, Department of Clinical Sciences, College of Veterinary Medicine North Carolina State University Raleigh North Carolina USA

**Keywords:** adverse effects, dog, extended‐release buprenorphine, injection site, sedation score, sex difference

## Abstract

This study evaluated the pharmacokinetics of an extended‐release buprenorphine formulation (Ethiqa XR) in dogs and explored potential sex differences. Twelve healthy intact beagles (6 males and 6 females) received a single subcutaneous injection of Ethiqa XR (0.2 mg/kg). Blood samples were collected up to 168 h post‐administration, and plasma buprenorphine concentrations were measured using liquid chromatography–tandem mass spectrometry. Vital signs, sedation, and nausea scores were recorded. Therapeutic plasma concentrations were sustained for approximately 60–90 h in both sexes, depending on the therapeutic threshold used (0.6 or 1.0 ng/mL). Although no significant differences in pharmacokinetics were detected, drug exposure and elimination were greater in females: median peak plasma buprenorphine concentrations (male: 1.6 ng/mL, female: 2.9 ng/mL); median terminal half‐life (male: 36.6 h, female: 24.7 h); area under the curve (AUC 0–12 h) (male: 12.8 h*ng/mL, female: 16.9 h*ng/mL); AUC (0–96 h) (male: 94.4 h*ng/mL, female: 137.7 h*ng/mL). Time to maximum concentrations were 24 h in both sexes. Higher buprenorphine concentrations were associated with decreased body temperature and heart rate in both sexes and positively correlated with nausea and sedation scores, but only in females. Ethiqa XR administration resulted in therapeutic plasma concentrations up to 90 h, suggesting it may be an alternative option for post‐operative pain control.

## 1 Introduction

Buprenorphine, a potent partial μ‐agonist opioid analgesic, is used for treatment of pain in companion and laboratory animals (Brodbelt et al. 1997; Watanabe et al. 2018, 2020). It demonstrates a ceiling effect on respiratory depression, has a relatively long duration of action, exerts minimal cardiovascular effects, and has a low tendency to induce vomiting, making it therapeutically favorable compared to other opioids (Martinez et al. 1997; Martin 1979; Yassen et al. 2008; Pergolizzi et al. 2010; Raffa et al. 2014; Nunamaker et al. 2014; Robertson and Taylor 2004; Andaluz et al. 2009). Although buprenorphine is not approved by the Food and Drug Administration (FDA) for use in dogs, it is commonly administered extra‐label in veterinary practice due to these advantages. However, “regular” buprenorphine HCL often requires multiple dosing a day to maintain adequate analgesia (Hansford et al. 2021).

Currently, an FDA‐indexed formulation of extended‐release (> 24 h efficacy) buprenorphine (XR‐Bup) injectable suspension is primarily limited to laboratory animals, including rodents, ferrets, rabbits, and non‐human primates. In major companion species, the only FDA‐approved option is transdermal extended‐buprenorphine solution (Zorbium; Elanco, US), however, this is labeled only for use in cats. To date, no FDA‐approved XR‐Bup formulation is available for dogs, despite evidence of XR‐Bup formulations demonstrating prolonged analgesic effects, sustained therapeutic plasma concentration, and minimal adverse events (Tomas et al. 2015; Barletta et al. 2018). However, these findings highlight that XR‐Bup may be clinically promising for the management of pain in the immediate postoperative period in both the hospital environment and at home. The first 72 h are considered critical for pain management after surgery, aligning with the inflammatory phase of wound healing. Analgesics should be provided for at least this period (Lascelles and Kirkby Shaw 2016; Leaper and Harding 1998). However, currently, there are no approved or practical opioid options for providing pain relief in the early “at home” period.

Ethiqa XR (Fidelis Animal Health, NJ, USA), an XR‐Bup product, is a lipid‐bound buprenorphine hydrochloride, suspended in medium‐chain fatty acid triglyceride oil. The lipid encapsulation slows the diffusion rate of the drug from the injection site and thereby prolongs the duration of the analgesic effect (Mishra et al. 2012; Bethune et al. 2001). Therefore, Ethiqa XR has the potential to provide effective post‐surgical analgesia and effectively cover the early “at home” period when post‐operative pain is greatest (first 72 h) (Nunamaker et al. 2014; Tomas et al. 2015; Barletta et al. 2018).

Sex‐related differences in the pharmacokinetics of buprenorphine have been documented across species, including respiratory effects in mice (Alhaddad et al. 2013), and dose‐dependent behavioral effects were more evident in female rhesus monkeys (Schwienteck et al. 2019). Female humans showed higher peak plasma concentrations of buprenorphine, norbuprenorphine, and NBUP‐3 glucuronide than in males (Moody et al. 2011), and females of reproductive age appeared more sensitive to the effects of opioids, likely due to higher brain μ‐opioid receptor concentrations (Zubieta et al. 1999).

The objective of this current prospective study was to evaluate the buprenorphine plasma concentration, pharmacokinetic profile, pharmacologic effects, and adverse effects in dogs following the single subcutaneous (SC) administration of Ethiqa XR (buprenorphine extended‐release injectable suspension, Fidelis Animal Health, NJ, USA) and to explore potential sex‐related differences. We hypothesized that the single SC administration of Ethiqa XR would result in sustained therapeutic buprenorphine plasma concentration for 72 h.

## 2 Material and Methods

### 2.1 Animals

Twelve purpose‐bred beagle dogs (6 intact males and 6 intact females) were obtained from an approved commercial vendor. The median age was 14 months (range 9–17), and the median weight was 9.6 kg (range 7.6–11.1). They were determined to be healthy based on physical examination (general, orthopedic and neurological) and blood examination (serum biochemical analysis and complete blood count). The dogs were maintained in a climate‐controlled facility with 12:12 light–dark cycle at the College of Veterinary Medicine, North Carolina State University. The temperature and humidity of the room were set at 23.0°C and 40%–50%, respectively. The dogs were individually housed from 48 h prior to buprenorphine administration through the end of the sampling period. Dogs were provided ad libitum access to water. The protocol was approved by the Institutional Animal Care and Use Committee (protocol number 25‐231).

### 2.2 Study Design/Treatment

This was a prospective study to evaluate the buprenorphine plasma concentration over time, including potential sex‐related differences, following SC administration in 12 healthy dogs.

Dogs were acclimatized for a minimum of 1 week before jugular catheter placement. To facilitate the blood collection, all dogs were sedated for catheter placement with 10 μg/kg dexmedetomidine (Dexdomitor; Zoetis Canada Inc., Quebec, CA) administered intravenously. A single lumen central venous catheter (Loveland, CO, USA) was placed aseptically with the routine Seldinger technique. Sedation was reversed with intramuscular (IM) injection of 0.1 mg/kg atipamezole (Antisedan; Zoetis, Kalamazo, MI, USA), and dogs were allowed 48 h to recover before the PK experiment started. The catheters were flushed during the study with 0.9% sterile saline and locked with heparinized saline to maintain patency at a minimum every 12 h. The catheter sites were monitored daily for any sign of inflammation or infection, and at the same time a bandage change was performed. None of the dogs had been exposed to buprenorphine within the previous 30 days prior to the experiment.

All dogs received a single SC injection of Ethiqa XR (0.2 mg/kg, 1.3 mg/mL, Fidelis Animal Health, NJ, USA), between the shoulder blades using a 20 G needle. All injections, scoring, and patient monitoring were performed by the same investigator (KT). The range of injection volume of Ethiqa XR was 1.2–1.7 mL. All dogs were fasted 12 h prior to Ethiqa XR administration.

The dose of Ethiqa XR selected for evaluation was based on previous work. In a study by Barletta et al. (2018), a mean plasma buprenorphine concentration of 1 ng/mL was confirmed for at least 72 h after the single SC injection of XR Bup (0.2 mg/kg, Animalgesic Labs), and significant antinociception was found over this time period. In other work, buprenorphine plasma concentrations > 0.6 ng/mL were found to be associated with therapeutic efficacy (no need for rescue analgesia) following ovariohysterectomy (Ko et al. 2011). Thus, 0.2 mg/kg was chosen as the dose to evaluate.

### 2.3 Physiological Parameter and Injection Site Monitoring

Respiratory rate (RR), heart rate (HR), and rectal temperature (T) were recorded at 0 (pre‐dose), 1, 2, 4, 6, 8, 12, 24, 36, 48, 60, 72, 96, 120, 144, 168 h following Ethiqa XR administration. Appetite, urination, water intake, defecation, and any gastrointestinal signs (nausea‐nauseous score, vomiting, regurgitation, diarrhea) were also recorded at each time point. The dog's regular dry food (Hill's Science Diet Adult Dry) was offered 2 h after drug administration, and again at 8 h. If the dogs were not interested in dry food for 24 h after drug administration, canned food (Hills Prescription Diet i/d Digestive Care Beef Stew) was offered with/without dry food.

Injection sites were evaluated at a minimum of 2 times a day for 10 days post‐administration of Ethiqa XR for signs of redness, swelling, tissue reaction, ulceration, and pain.

### 2.4 Nausea Scoring

The presence and degree of nausea were recorded at 0 (pre‐dose), 1, 2, 4, 6, 8, 12, 24, 36, 48, 60, 72, 96, 120, 144, 168 h following drug administration. Nausea scoring (NSC) was performed with reference to previously published methods (Kenward et al. 2014). At each time point, visual analog scales (VAS) from 0 to 100 mm (100 = worst possible nausea/vomiting). Signs such as food aversity, lip smacking salivation, abdominal discomfort/restlessness, and vomiting were signs that were evaluated to determine where on the 100 mm line the degree of nausea was marked.

### 2.5 Sedation Scoring

Baseline (time 0) sedation score (SSC) was recorded during the acclimation period and before drug administration on the dosing day. After drug administration, SSC were recorded at 1, 2, 4, 6, 8, 12, 24, 36, 48, 60, 72, 96, 120, 144, 168 h timepoint by the same investigator (KT). Sedation was assessed using the seven‐point sedation scale validated in dogs by Wagner et al. (2017) with one modification (see scoring system provided in File S1). As can be seen from the sedation score scale (File S1), resistance when placed in lateral recumbency was also part of the original scale. However, this was not applicable in these beagles, as they were trained for this procedure and showed no resistance at any time during the study. Therefore, “resistance to being placed in lateral recumbency” was removed from the scale, and our maximum sedation score was 18.

### 2.6 Sample Collection

Blood samples (3 mL) were collected at 0 h (pre‐dose/negative control) and at 0.083, 0.25, 0.5, 0.75, 1, 2, 4, 6, 8, 12, 24, 36, 48, 60, 72, 96,120, 144, and 168 h following administration of Ethiqa XR. An initial 2–3 mL “clearance sample” was collected and set aside, followed by collection of the blood sample (3 mL) to be used for measuring plasma concentration of buprenorphine and the “clearance sample” was returned to the dog, flushed with 0.9% sterile saline at minimum every 12 h. If the interval between sampling was longer than 2 h, the catheter was flushed with heparinized saline. Blood samples were transferred into tubes containing lithium heparin anticoagulant (BD Vacutainer; Franklin Lakes, NJ, USA) and placed on ice. The blood samples were centrifuged for 15 min at 2058 × *g* within 1 h after sampling to collect plasma. Then, plasma samples were stored at −80°C until analysis. The sampling catheters were removed after the final blood sample collection.

### 2.7 Buprenorphine Assay

#### 2.7.1 Chemical and Reagents

All reagents except ammonium hydroxide were LC/MS grade. Formic acid, acetonitrile (ACN), methanol (MeOH), acetic acid, 29% ammonium hydroxide, and o‐phosphoric acid were supplied by Fisher Chemical (NJ, USA). USP buprenorphine hydrochloride reference standard (CAS: 53152‐21‐9) was purchased from Sigma‐Aldrich (Sigma‐Aldrich RTC, Laramie, WY, USA). A Synergy water purification system (Millipore) was used to produce ultrapure water. Analysis of buprenorphine was carried out via ultra‐performance liquid chromatography (UPLC) and tandem mass spectrometric (MS/MS) detection (Waters Corporation, Milford, MA). The UPLC‐MS/MS system consisted of a Waters Acquity UPLC I class Binary Solvent Manager, Acquity UPLC Sample Manager FTN, and a Xevo TQD tandem mass spectrometer (Waters Corporation, Milford, MA).

#### 2.7.2 Sample Preparation

Buprenorphine was extracted from canine plasma samples using a validated assay reported by Enomoto et al. 2022. Plasma (350 μL) was pretreated by mixing with 350 μL of 5% phosphoric acid in water in a clean glass tube (disposable culture tubes, borosilicate glass, VWR) and vortexed thoroughly prior to solid phase extraction. Solid phase extraction was performed by using Oasis MCX 96 well μElution plate (Water Corporation, Milford, MA). The plate was prepared by first conditioning it with 200 μL of MeOH followed by 200 μL of ultrapure water. Then, 700 μL of pretreated plasma was loaded on the MCX μElution plate and passed through the plate by using a positive pressure manifold, Otto Specialist (Waters Corporation, Milford, MA). The plate was then washed with 200 μL of 0.2% acetic acid prepared in water followed by 200 μL 100% MeOH under positive pressure controlled by the Otto Specialist. The analyte was eluted into a clean 96 well collection plate (700 μL round 96 well samples plate, Waters Acquity UPLC, Water corporation, Milford, MA) with addition of 50 μL of elution solution (ACN: MeOH: 29% ammonium hydroxide = 57%: 38%: 5%), and 50 μL of water was added to the eluent and mixed thoroughly before analysis. Samples, 2 μL, were injected into the UPLC‐MS/MS to measure the buprenorphine concentrations.

A calibration curve was created by using blank canine plasma (drug‐free) and was fitted with a weighted (1/concentration) linear equation by using Targetlynx software. Calibration ranges of 0.01–50 ng/mL were linear with a coefficient of determination, *R* ^2^, > 0.995. Each calibration standard concentration could be back‐calculated to within 15% of the nominal concentration. Blank canine plasma (drug‐free) and the quality controls (0.3, 3, 7, and 30 ng/mL) were injected with every batch. Blank canine plasma did not include buprenorphine. Inter‐day precision and accuracy are provided in File S2. The limit of detection (LOD) and limit of quantification (LOQ) were 0.01 ng/mL and 0.1 ng/mL, respectively, based on signal to noise ratio (LOD: a peak area > 3 times blank peak area and LOQ: a peak area > 5 times the blank peak area), inter‐day precision and accuracy, and chromatogram, respectively.

#### 2.7.3 UPLC‐MS/MS Conditions

Chromatographic separation was performed by a gradient elution with the ACQUITY UPLC HSS T3 1.8 μm column (2.1 × 100 mm) and VanGuard pre‐column (Waters Corporation, Milford, MA). The mobile phase solvents consisted of 0.1% formic acid prepared in water (A) and 0.1% formic acid prepared in ACN (B). Flow rate was 0.4 mL/min and run time was set as 5 min. The gradient mobile phase conditions were programmed as 85% of A and 15% of B for the first 2.5 min, then changed linearly to 10% of A and 90% of B from 2.5–4.0 min, back to 85% of A and 15% of B from 4.0–5.0 min to re‐equilibrate at the initial conditions. The column and autosampler temperature were maintained at 40°C and 10°C, respectively. The positive electrospray ionization mode (ESI (+)) was used with the multiple reactions monitoring (MRM) and the capillary and cone voltages on the MS tune page source were 0.7 kV and 84 V, respectively. On the MS tune page, the source desolvation temperature was 500°C. The source of desolvation gas flow and the cone gas flow were 800 L/h and 50 L/h, respectively. The MS file cone voltage setting was 82 V and collision energy settings were 48 V and 50 V. Nitrogen was used as the desolvation and cone gases and argon as the collision gas. Quantification was performed using the transition Parent (m/z): 468.2681 and Daughter (m/z): 83.7420 and 100.9775. The retention time was 2.41 min consistently.

### 2.8 Pharmacokinetic Analysis

Pharmacokinetic analysis was conducted using pharmacokinetic software (Phoenix WinNonLin ver 8.5, Certara St. Louis, MO, USA). Only values above LOD were included in the analysis. Values below LOQ and above LOD were converted to LOQ/2 (0.05 ng/mL) for this analysis. Non‐compartmental analysis was performed to estimate the pharmacokinetic parameters including the elimination rate constant (Lambda_Z), terminal half‐life (HL__Lambda_z_), the time to maximum concentration (*T*_max_), the maximum concentration (*C*_max_), the area under the curve from time zero to 12 h time point (AUC_0–12_), the area under the curve from time zero to 24 h time point (AUC_0–24_), the area under the curve from time zero to 72 h time point (AUC_0–72_), the area under the curve from time zero to 96 h time point (AUC_0–96_), the area under the curve from time zero to the last time point (AUC_last_), the area under the curve from time zero to infinity (AUC_inf_), and mean residence time (MRT_inf_), which were calculated using the linear log trapezoidal method and standard non‐compartmental equations (Rosembaum 2011).

### 2.9 Statistical Analysis

All statistical analyses were performed using R (version 4.5.1, released 2025‐06‐13). Wilcoxon rank‐sum test was conducted to explore the gender group differences for the 11 pharmacokinetic variables (Lambda_Z, HL__Lambda_z_, *T*_max_, *C*_max_, AUC_0–24_, AUC_0–72_, AUC_0–96_, AUC_last_, AUC_inf_ and MRT_inf_). They were treated as continuous variables and the median was used as the estimator for each sex group, followed by a two‐independent‐group nonparametric test. Differences were considered significant at *p* < 0.05. Cliff's delta effect size and the magnitude scale level of the effect size for each pharmacokinetic variable were also calculated (Cliff 1993).

Repeated‐measures correlations (within subject) between buprenorphine concentration and T^o^F, HR, RR, NSC, and SSC were analyzed using the R package rmcorr, following Bland and Altman (1995). The interpretation of the correlation is whether an increase in buprenorphine concentration within an individual is associated with an increase or decrease in another variable (HR, RR, T, NS, SSC). Due to repeated testing, we applied the Benjamini–Hochberg (BH) adjustment to all original *p*‐values. We used *p* < 0.05 to declare statistically significant findings in the analysis.

## 3 Results

The median ages for male and female dogs were 14 months (14–17) and 14 months (9–17), respectively. Median body weight was 10.7 kg (9.6–11.1) in males and 8.2 kg (7.6–10.6) in females (Tables 1 and 2).

**TABLE 1:** Physiologic variables (age, body weight, vitals, NSC, SSC) over time and their correlation with buprenorphine concentration (ng/mL) in males.

Timeline/h	Age	BW/kg	HR/min	T (°F)	RR/min	NSC	SSC	Buprenorphine c. (ng/mL)
0	14 month	10.7	108.6	101.0	23.3	0	0	0
1			100.6	100.9	24.6	1.6	0.4	0.4
2			93.3	100.4	23.3	11.7	1	0.7
4			89.3	99.8	21.3	8.3	2.2	1.1
6			88.6	99.4	20.6	5.8	1.8	1.3
8			94	99.7	23.3	20.8	1.6	1.4
12			85.3	99.7	22	12.5	1.6	1.3
24			83.3	99.9	24	3.3	1.2	2.7
36			94	100.3	24.6	6.6	0.2	1.9
48			90.3	100.3	27	1.6	0.2	1.4
60			102	100.7	24.6	0	0	1.0
72		10.1	107.6	100.6	26	0	0	0.9
96			110	101.3	24.6	16.6	0	0.7
120			116	101.3	25.3	0	0	0.4
144			122	101.7	27.3	16.6	0	0.2
168			125.6	101.7	24.6	0	0	0.1

Abbreviations: BW, body weight; HR, heart rate; NSC, nauseous score; RR, respiratory rate; SSC, sedation score; T, rectal temperature.

**TABLE 2:** Physiologic variables (age, body weight, vitals, NSC, SSC) over time and their correlation with buprenorphine concentration (ng/mL) in females.

Timeline/h	Age	BW/kg	HR/min	T (°F)	RR/min	NSC	SSC	Buprenorphine c. (ng/mL)
0	14 month	8.2	106	100.8	22.6	0	0	0
1			110	101.2	23.3	3.3	0	0.7
2			102	100.8	24	43.3	0.7	1.6
4			92	99.8	19.3	28.3	3.5	1.2
6			91.3	99.3	20.6	6.6	2.7	2.2
8			92	99.65	22.6	21.6	2	1.6
12			90	100.0	24	8.3	1.8	2.1
24			86	100.05	24	5	1.8	2.3
36			97.3	100.5	24	20	1	2.0
48			95.3	100.6	24	16.6	0.3	1.8
60			103.3	100.7	25.3	16.6	0.2	1.3
72		8.1	102.6	100.8	24.6	18.3	0.2	1.0
96			117.3	101.6	25.3	0	0.2	0.5
120			124.3	101.6	25.3	0	0	0.2
144			120.3	101.8	25.3	0	0	0.1
168			114	101.85	24	0	0	0.1

Abbreviations: BW, body weight; HR, heart rate; NSC, nauseous score; RR, respiratory rate; SSC, sedation score; T, rectal temperature.

All dogs completed the study. Two dogs developed mild skin irritation around the ventral neck, secondary to the collar used to prevent interference with the catheter site (BiteNot Collar, KVP International LLC, McKinney, TX), which were resolved with local treatment using chlorhexidine and Silver Sulfadiazine Cream (SSD cream, Ascend Laboratories LLC, Parsippany, NJ 07054). One dog (female 6) showed signs of pseudogravidity and another dog (female 4) was in proestrus at the start of the study.

### 3.1 Rectal Temperature, Pulse Rate, Respiratory Rate

The normal range for monitored variables was defined as RR: 12–30 breaths/min, HR: 70–140/min, and T: 99.5°F–102.5°F (Haskins et al. 2005).

**FIGURE 1:**
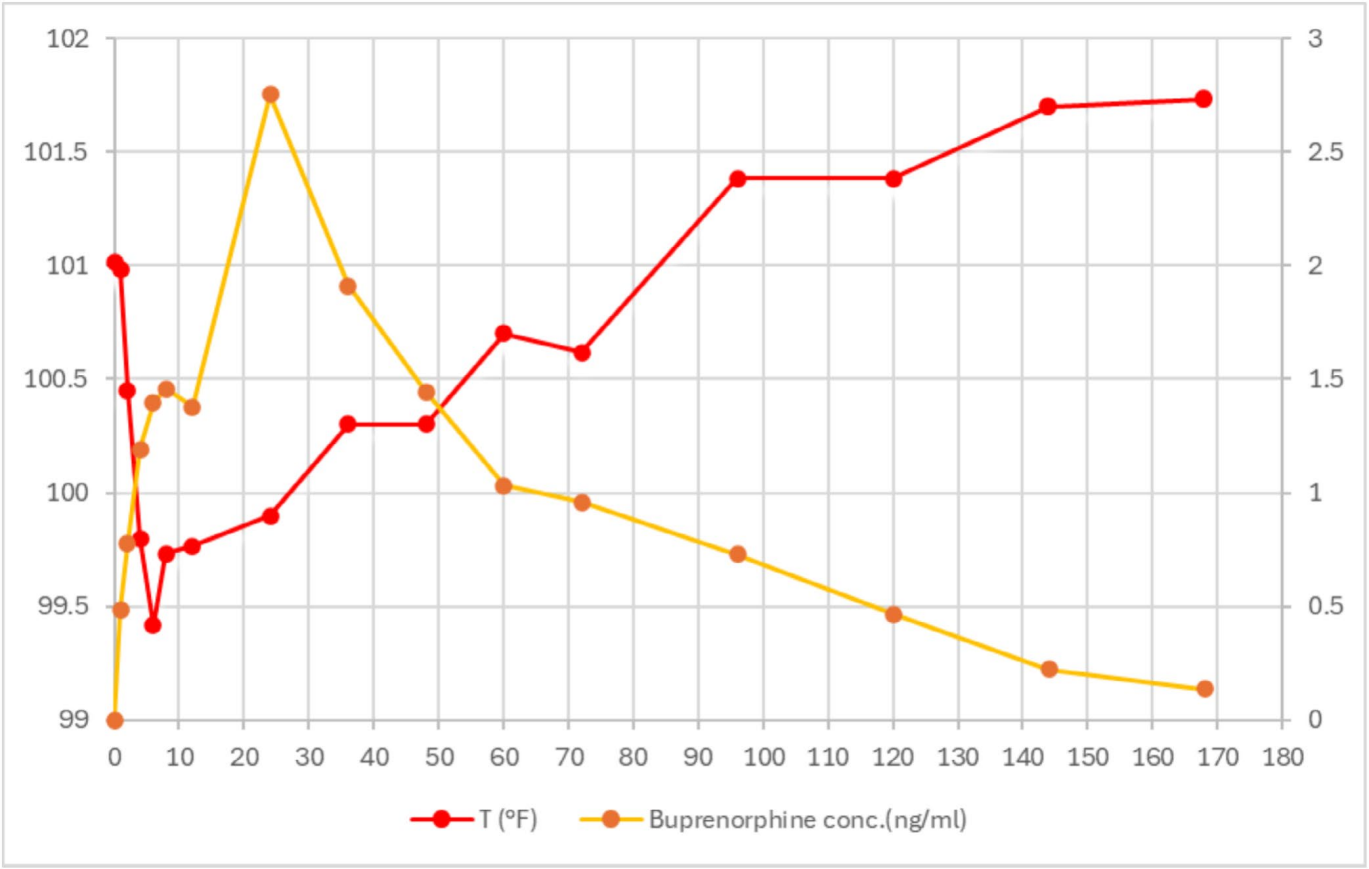
Mean buprenorphine plasma concentrations (ng/mL) and concurrent rectal temperature (T, °F) over time (h) in males after administration of a single subcutaneous dose of 0.2 mg/kg Ethiqa XR.

**FIGURE 2:**
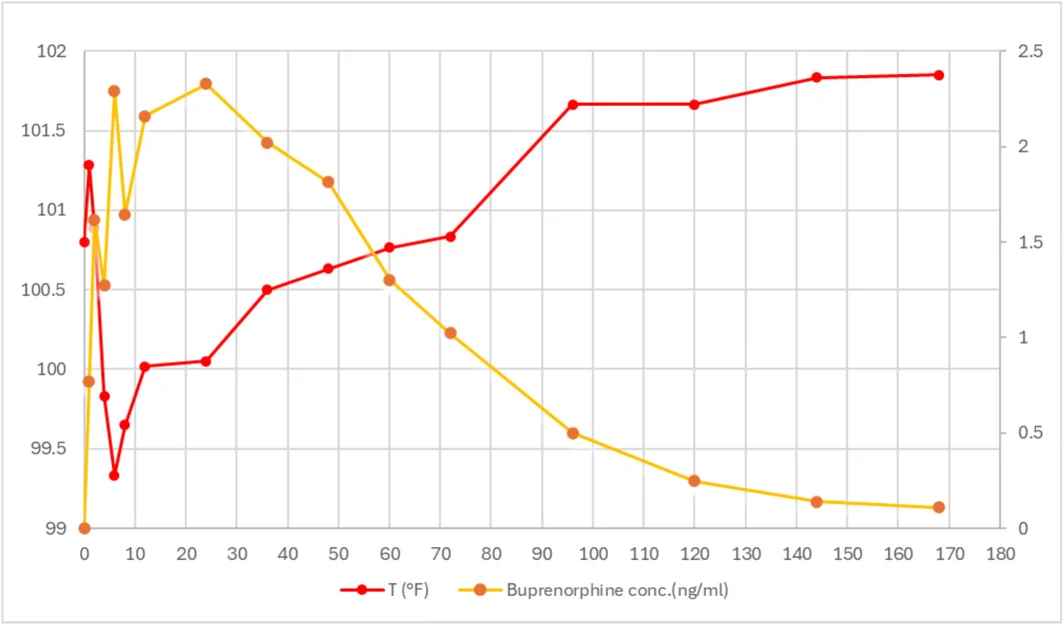
Mean buprenorphine plasma concentrations (ng/mL) and concurrent rectal temperature (T, °F) over time (h) in females after administration of a single subcutaneous dose of 0.2 mg/kg Ethiqa XR.

Overall, rectal temperature (T, ^o^F) appeared to decrease following the administration of Ethiqa XR and gradually returned to normal (Tables 1 and 2). Based on 99.5°F degrees being the threshold for hypothermia, seven dogs were considered hypothermic (4 males, 3 females) between 4‐ and 36 h post‐injection, with the lowest mean temperature (99.0°F ± 0.48) at 6 h following Ethiqa XR administration (Figures 1 and 2). Significant negative correlations were observed between buprenorphine concentration and T^o^F in both males (*r* = −0.56, pBH < 0.001) and females (*r* = −0.59, pBH < 0.001) (Table 3).

**TABLE 3:** Repeated‐measures correlation results between buprenorphine concentration and physiological variables, stratified by sex.

Outcome	Sex	*r*	95% CI	*p*	pBH
HR	M	−0.61	(−0.73, −0.46)	9.6 × 10^−11^★	4.8 × 10^−10^★
HR	F	−0.65	(−0.76, −0.5)	6.6 × 10^−12^★	6.6 × 10^−11^★
RR	M	−0.08	(−0.28, 0.12)	4.3 × 10^−1^	4.8 × 10^−1^
RR	F	−0.16	(−0.36, 0.05)	1.4 × 10^−1^	1.9 × 10^−1^
T	M	−0.56	(−0.69, −0.40)	5.0 × 10^−9^★	1.2 × 10^−8^★
T	F	−0.59	(−0.71, −0.43)	1.7 × 10^−9^★	5.6 × 10^−9^★
NSC	M	−0.004	(−0.21, 0.20)	9.7 × 10^−1^	9.7 × 10^−1^
NSC	F	0.24	(0.03, 0.42)	2.6 × 10^−2^★	4.3 × 10^−2^★
SSC	M	−0.09		3.69 × 10^−1^	4.61 × 10–1
SSC	F	0.51		2.68 × 10^−7^	5.35 × 10–7★

*Note:* Significant correlations after Benjamini–Hochberg (BH) correction are marked with “★”. Repeated‐measures correlations were performed separately within each sex group. The column *r* represents the estimated correlation value, positive *r* values indicate a positive correlation (same direction) between the two variables, while negative values indicate a negative correlation (opposite direction), df indicates the degrees of freedom in the analysis, *p*‐value is the unadjusted *p*‐value from the analysis, followed by the 95% confidence interval of the estimated correlation value (*r*).

Abbreviations: HR, heart rate; NSC, Nauseous score; RR, Respiratory rate; SSC, Sedation score; T, Rectal temperature.

**FIGURE 3:**
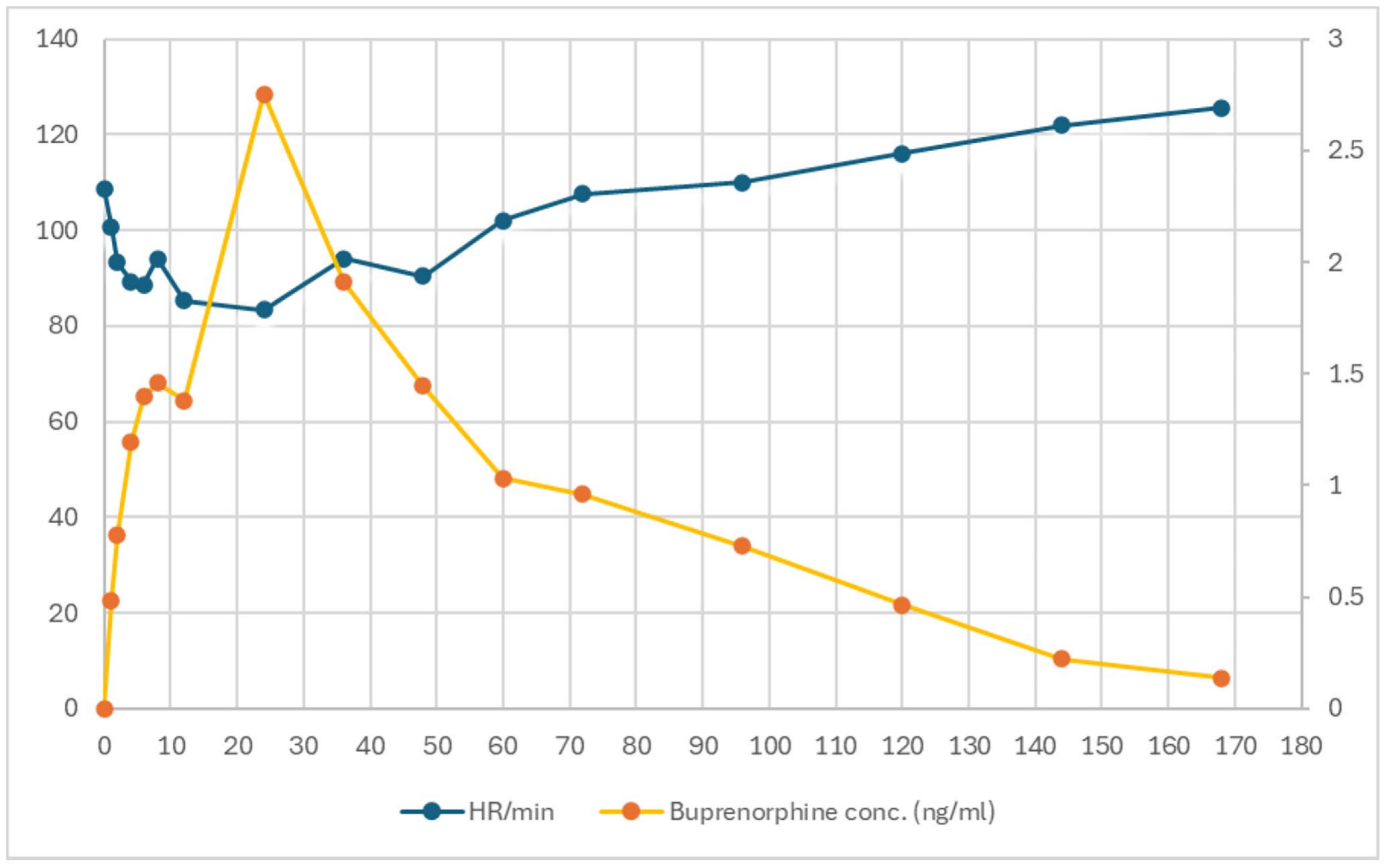
Mean buprenorphine plasma concentrations (ng/mL) and concurrent heart rate (HR) over time (h) in males after administration of a single subcutaneous dose of 0.2 mg/kg Ethiqa XR.

**FIGURE 4:**
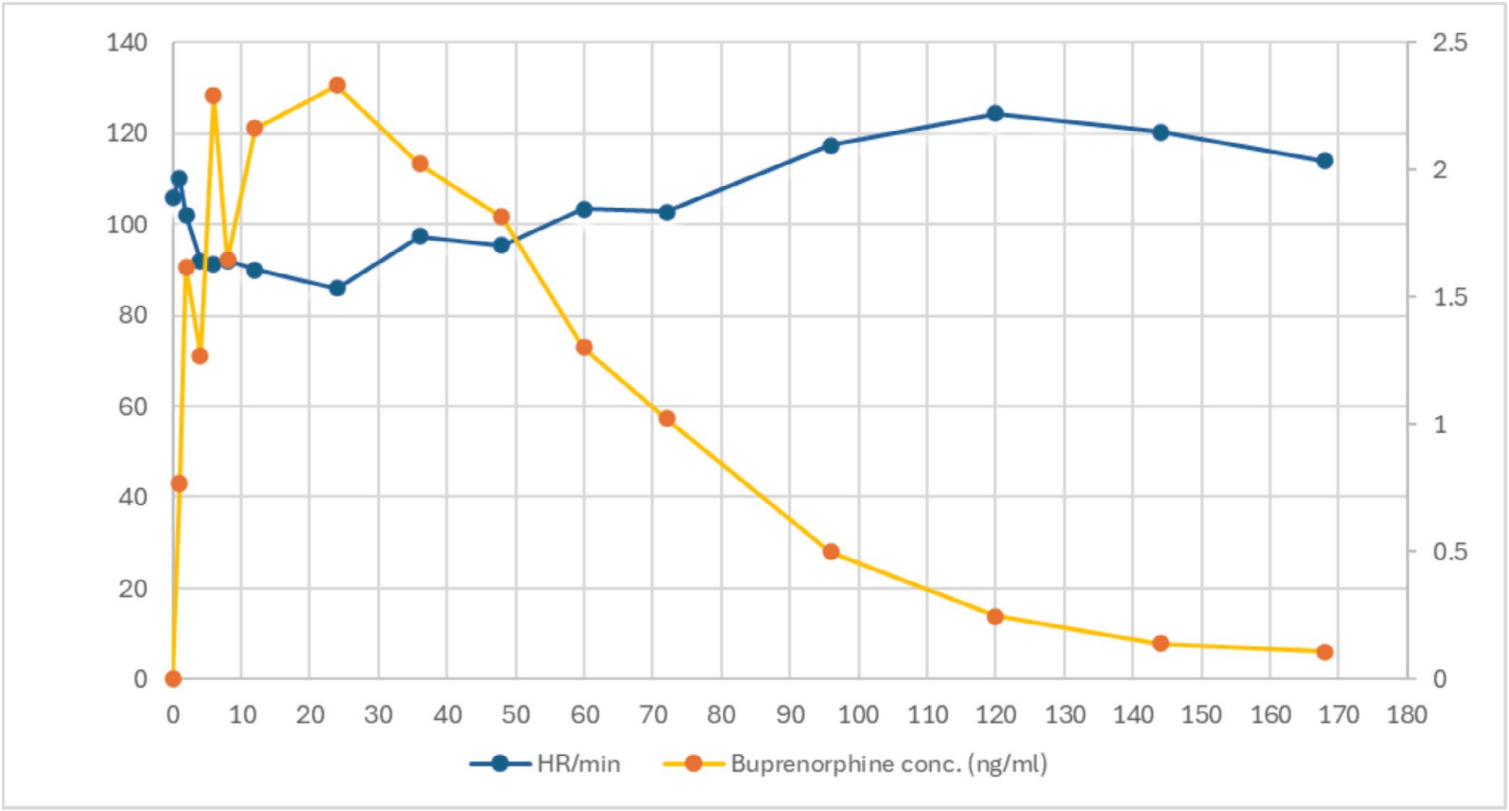
Mean buprenorphine plasma concentrations (ng/mL) and concurrent heart rate (HR) over time (h) in females after administration of a single subcutaneous dose of 0.2 mg/kg Ethiqa XR.

Generally, the HR decreased post‐injection at 2 h and returned to baseline (HR: 116) 96 h, but only one female dog (# 4) was considered bradycardic, with HR 68 bpm at 12‐ and 24‐h post buprenorphine injection. The mean HR was 103 ± 16 bpm, range 68–140, median 100 (Figures 3 and 4). Significant negative correlations were observed between buprenorphine concentration and HR in both males (*r* = −0.61, pBH < 0.001) and females (*r* = −0.65, pBH < 0.001) (Table 3).

Respiratory rate versus time (hours) is shown in Tables 1 and 2. The mean respiratory rate (RR) was 24 ± 3 bpm, range 16–30, median 24. No significant correlation was observed between buprenorphine concentration and RR in either males (*r* = −0.08, pBH = 0.48) or females (*r* = −0.16, pBH = 0.19) (Table 3).

There was no significant mean difference between females and males in RR and HR throughout the study.

### 3.2 Sedation Score

**FIGURE 5:**
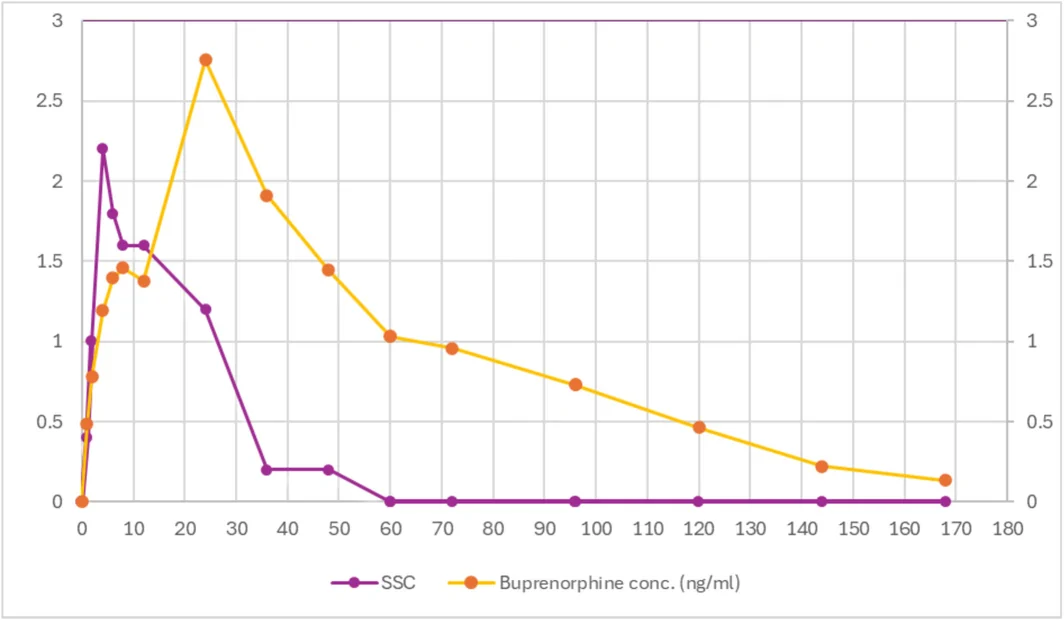
Mean buprenorphine plasma concentrations (ng/mL) and concurrent sedation score (SSC) over time (h) in males after administration of a single subcutaneous dose of 0.2 mg/kg Ethiqa XR.

**FIGURE 6:**
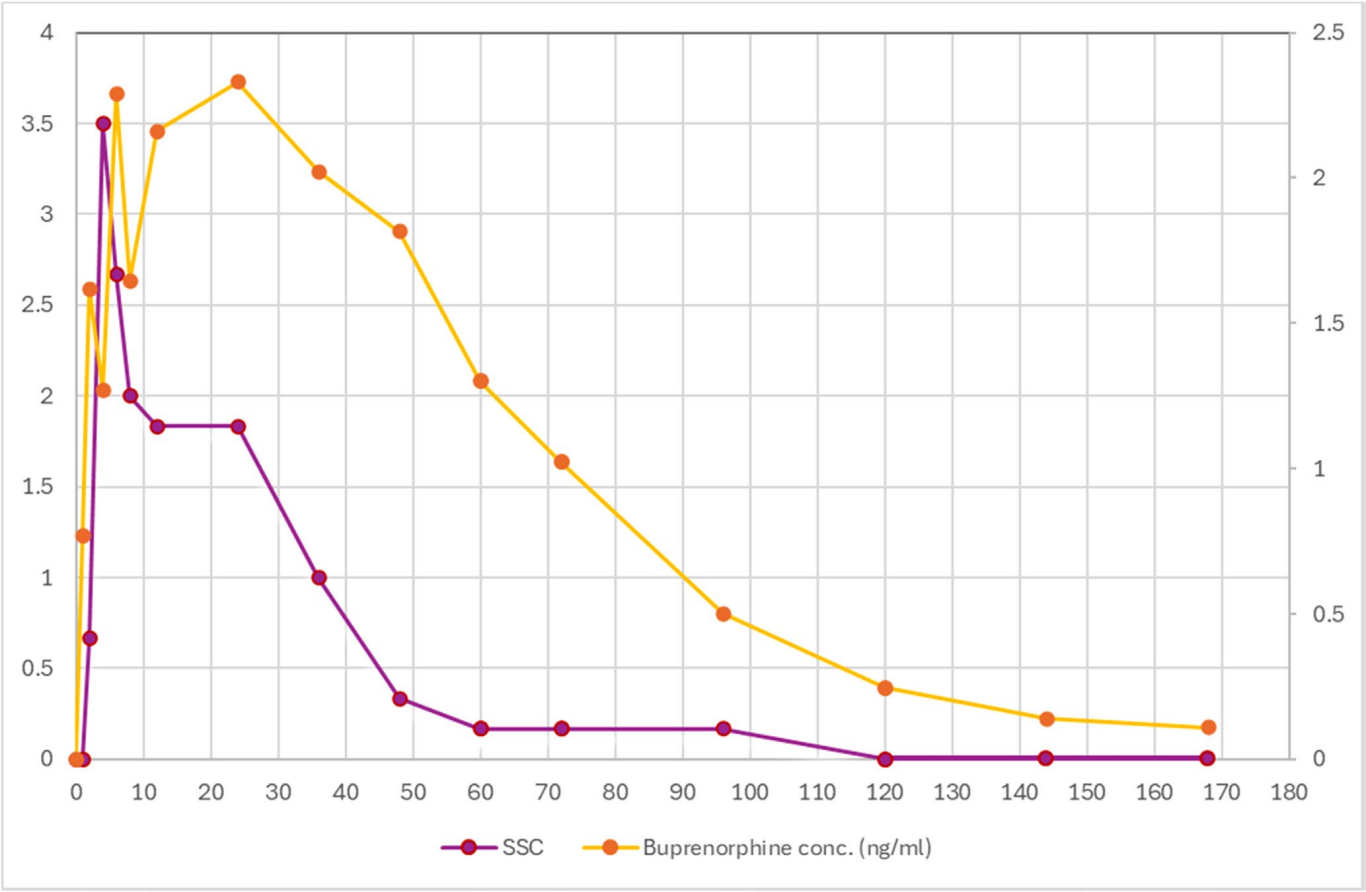
Mean buprenorphine plasma concentrations (ng/mL) and concurrent sedation score (SSC) over time (h) in females after administration of a single subcutaneous dose of 0.2 mg/kg Ethiqa XR.

The SSC versus time (hours) is shown in Figure 5 (males) and Figure 6 (females), and Table 1 (males) and Table 2 (females). Dogs appeared the most sedated at 4 and 6 h after buprenorphine administration compared to baseline values. Across all dogs combined, SSC subsequently decreased and did not correlate with buprenorphine plasma concentration. However, there was a significant positive correlation found between buprenorphine concentration and SSC in females (*r* = 0.51, pBH < 0.001), while no association was observed in males (*r* = −0.10, pBH = 0.46) (Table 3, Figures 5 and 6).

### 3.3 Injection Site

No injection site reactions (erythema, heat, pain, or swelling) were observed at the time of the administration and up to 10 days after Ethiqa XR administration for all dogs.

### 3.4 Gastrointestinal Signs

**FIGURE 7:**
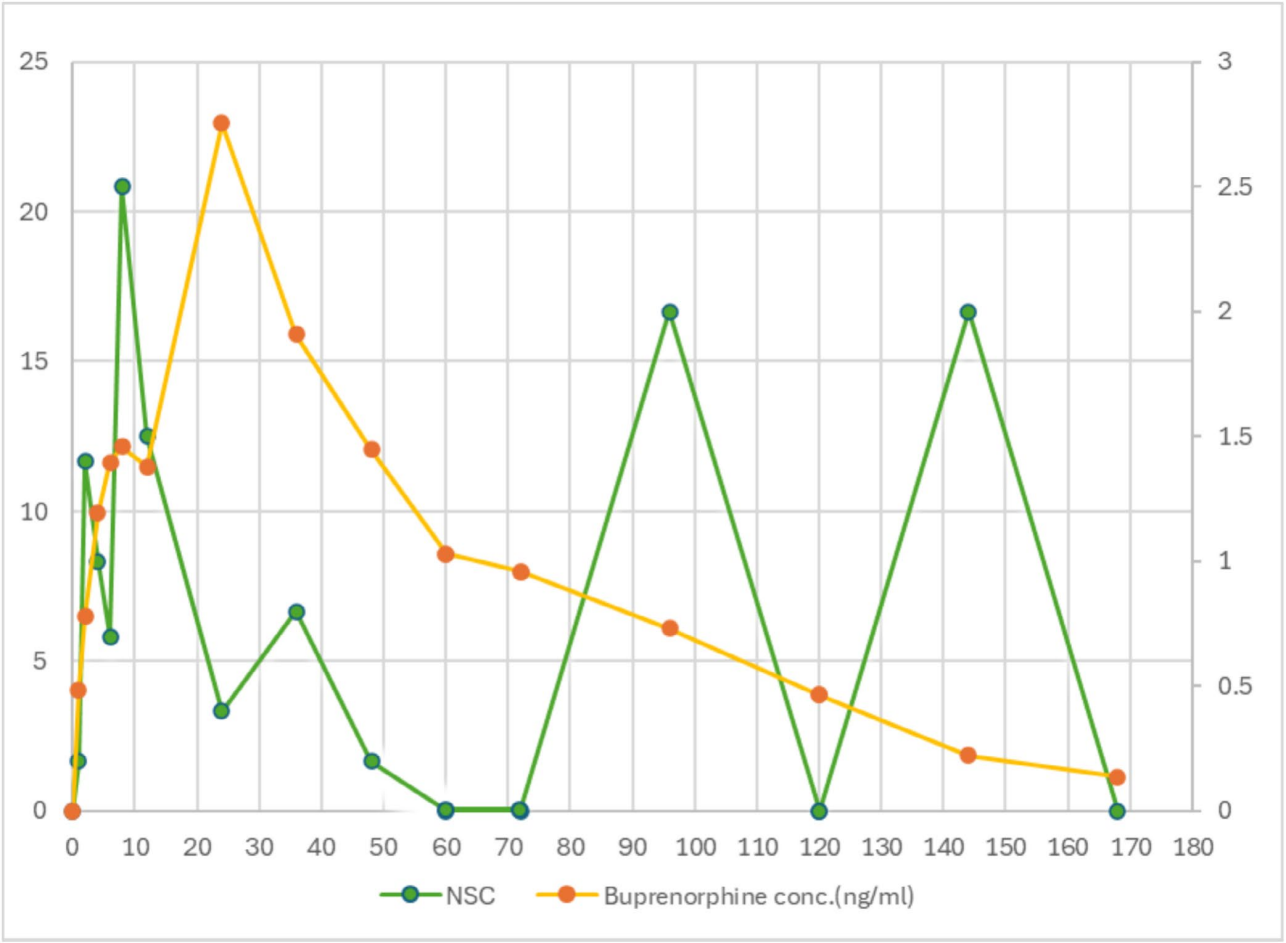
Mean buprenorphine plasma concentrations (ng/mL) and concurrent nauseous score (NSC) over time (hours) in males after administration of a single subcutaneous dose of 0.2 mg/kg Ethiqa XR.

**FIGURE 8:**
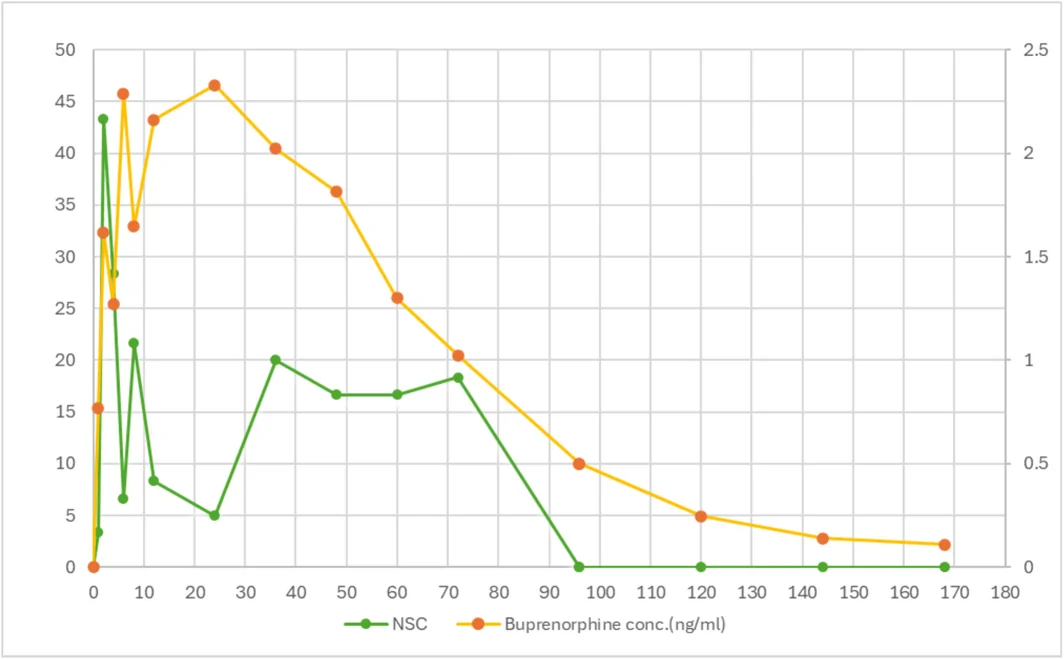
Mean buprenorphine plasma concentrations (ng/mL) and concurrent nauseous score (NSC) over time (h) in females after administration of a single subcutaneous dose of 0.2 mg/kg Ethiqa XR.

All of the dogs showed signs (hypersalivation, anorexia) of nausea at some point during the study (Tables 1 and 2). Vomiting occurred in 4 dogs (33.3%)—1 male (16.6%) and 3 females (50%). Across all dogs, the mean timespan over which vomiting occurred (mean ± SD (range) was 47.6 ± 38.6 h (2–120 h)). In the female dogs, vomiting occurred 2 to 3 times between 2 and 90 h post‐Ethiqa XR administration (mean ± SD, 30 ± 19.7 h), while the one male dog vomited 4 times between 8 h and 120 h following drug administration. A significant positive correlation with buprenorphine plasma concentration was observed in females (*r* = 0.24, pBH = 0.04), but not in males (*r* = −0.004, pBH = 0.97) (Table 3) (Figures 7 and 8).

All the dogs experienced reduced appetite at some point. The anorexia/hyporexia began in most cases between 2 and 4 h timepoint after administration of Ethiqa XR. The overall mean duration of anorexia/hyporexia was almost 3 days (67.5 ± 49.8, range 1–164, median 69 h); however, the males appeared more affected, with a mean duration of anorexia/hyporexia of almost 4 days (90 h ± 52.4, range 24–164, median 79 h) compared with < 2 days (44.5 h ± 37.8, range 1–92, median 50 h) in females.

Increased frequency of defecation (range 5–10 times) was observed in 8 of 12 dogs at the first 48 h. The mean duration of diarrhea or soft feces (mean ± SD (range)) for all dogs was almost 3 days (67.2 ± 55.1 h, range 1–162 h, median 48 h). Males were affected for a longer period, over 3.5 days (87.6 ± 68 h, 24–162 h, median 84 h), while females were affected approximately for 2 days (46.8 ± 32.5 h, 1–94 h, median 47).

An unusual sign was noted in the same female that showed tenesmus; she strained to urinate once shortly after the episode of straining to defecate at the 2 h timepoint. Her urination subsequently normalized, and no further urinary abnormalities were observed.

Overall, although GI signs continued in several dogs for up to a week, most returned to normal within 3–4 days.

### 3.5 Buprenorphine Concentrations in Plasma

**FIGURE 9:**
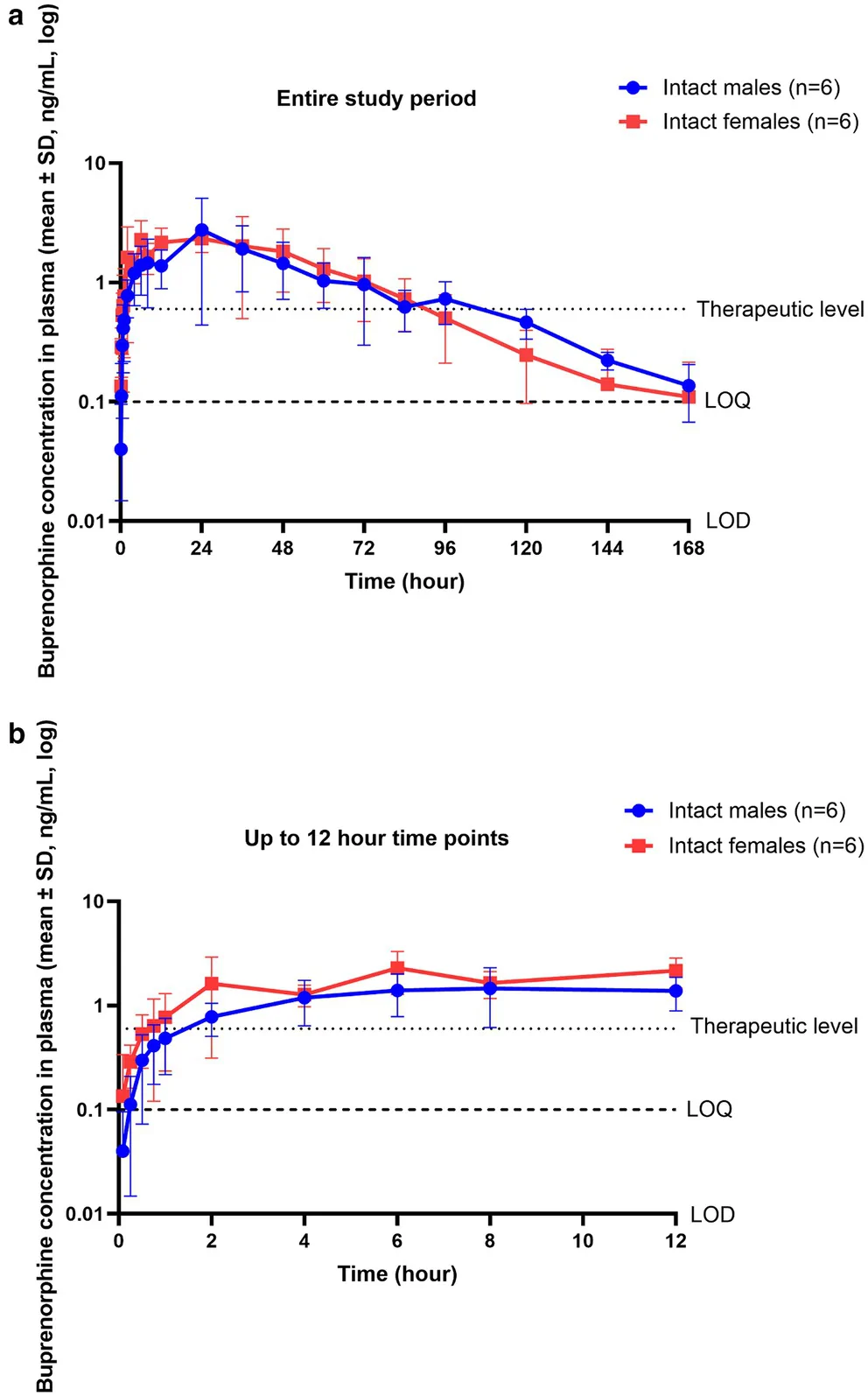
(a, b) Buprenorphine plasma concentration (mean ± standard deviation, ng/mL) versus time (hours) curve after a single subcutaneous administration of extended‐release buprenorphine (Ethiqa XR; 0.2 mg/kg) in intact male dogs (*n* = 6) and intact female dogs (*n* = 6). The dashed line represents the limit of quantification (LOQ, 0.1 ng/mL) of the assay. All data above the limit of detection (LOD, 0.01 ng/mL) were used for this figure. Therapeutic level: The plasma concentration (0.6 ng/mL, dotted line) that has been reported to be associated with analgesic effects in dogs for ovariohysterectomy (Ko et al. 2011). (a) Entire study period. (b) Up to 12 h.

The plasma concentration of buprenorphine (mean ± SD) versus time (hours) curves after a single SC administration of Ethiqa XR are shown in Figure 9a. The plasma concentrations of buprenorphine versus time curves for the initial 12‐h period are displayed in Figure 9b. Individual figures for the entire study period and up to 8‐h time points are shown in Figure S1a–d. Maximum plasma buprenorphine concentrations occurred at 24 h and were 1.6 ng/mL (1.2–6.8) (median and range) and 2.9 ng/mL (2.3–5.0) for the male group and the female group, respectively. All male dogs and 5 out of 6 females had detectable buprenorphine concentrations (> 0.01 ng/mL) at the last time point, 168 h (7 days). In males, plasma buprenorphine concentrations remained above 0.6 ng/mL for a median of 96 h (range, 60–96 h) and above 1 ng/mL for a median of 60 h (range 36–96 h) following administration. In females, buprenorphine plasma concentrations were above 0.6 ng/mL and 1 ng/mL for a median of 84 h (range, 48–96 h) and for a median of 72 h (range of 24–84 h) after administration, respectively. Inter‐dog variability in buprenorphine concentrations in plasma was observed in both groups.

### 3.6 Pharmacokinetic Parameters

Pharmacokinetic parameters for females and males are presented in Table 4. The non‐parametric comparison in pharmacokinetic variables between male and female groups is shown in Table 5. In the female group, there was a tendency toward higher buprenorphine exposure, evidenced by larger median buprenorphine plasma concentrations (female: 2.9 ng/mL vs. male: 1.6 ng/mL) and larger median drug exposure (area under the plasma concentration‐time curve, AUC) (AUC: female > male). The difference was most noticeable during early time periods, specifically at 0–12 h (female: 16.9 vs. male: 12.8 h*ng/mL) and 0–24 h (female: 43.8 vs. male: 29.2 h*ng/mL). Additionally, a faster median drug elimination was seen in females compared to males [HL__Lambda_z_: female (24.7 h) < male (36.6 h), MRT_inf_: female (47.0 h) < male (62.3 h)]. However, statistically significant differences were not found in any pharmacokinetic variables between females and males.

**TABLE 4:** Plasma pharmacokinetic parameters (median and range) estimated by using non‐compartmental analysis after single subcutaneous (0.2 mg/kg) administration of extended‐release buprenorphine (Ethiqa XR) in 6 intact male and 6 intact female healthy beagles.

Parameters	Units	Intact male, *n* = 6	Intact female, *n* = 6
Lambda_z	1/h	0.03 (0.01–0.05)	0.03 (0.02–0.04)
HL__Lambda_z_	h	36.6 (13.6–61.2)	24.7 (18.1–38.9)
*T*_max_	h	24.0 (8.0–36.0)	24.0 (6.0–36.0)
*C*_max_	ng/mL	1.6 (1.2–6.8)	2.9 (2.3–5.0)
AUC_0–12_	h*ng/mL	12.8 (6.1–22.2)	16.9 (14.1–30.3)
AUC_0–24_	h*ng/mL	29.2 (17.1–74.3)	43.8 (35.5–67.0)
AUC_0–72_	h*ng/mL	79.1 (63.0–218.7)	116.1 (65.6–216.5)
AUC_0–96_	h*ng/mL	94.4 (76.4–250.0)	137.7 (71.2–240.3)
AUC_last_	h*ng/mL	124.8 (100.1–279.7)	159.0 (77.9–252.4)
AUC_inf_	h*ng/mL	126.6 (117.6–280.7)	163.0 (79.8–253.3)
AUC_extrap_	%	4.1 (0.4–16.2)	1.6 (0.4–6.1)
MRT_inf_	h	62.3 (48.6–95.7)	47.0 (38.5–65.0)

*Note:* The samples below the limit of quantification (0.1 ng/mL) and above the limit of detection (0.01 ng/mL) of the analytical assay were converted to 0.05 ng/mL for this pharmacokinetic analysis.

Abbreviations: AUC_0–12_, area under the curve from time zero to 12 h time point; AUC_0–24_, area under the curve from time zero to 24 h time point; AUC_0–72_, area under the curve from time zero to 72 h time point; AUC_0–96_, area under the curve from time zero to 96 h time point; AUC_extrap_, extrapolation percent of the area under the curve; AUC_inf_, area under the curve from time zero to infinity; AUC_last_, area under the curve from time zero to the last time point; *C*_max_, maximum concentration; HL_Lambda_z, terminal half‐life; Lambda_z, elimination rate constant; MRT_inf_: mean residence time; *T*_max_, time to maximum concentration.

**TABLE 5:** Non‐parametric comparison of pharmacokinetic variables between male and female groups using Wilcoxon rank‐sum test.

Parameters	Median (male)	Median (female)	*p*	Cliff's *δ*	Magnitude
Lambda_z	0.03	0.03	0.94	0.06	Negligible
HL__Lambda_z_	36.6	24.7	0.94	−0.06	Negligible
*T*_max_	24	24	0.59	−0.19	Small
*C*_max_	1.6	2.9	0.30	0.39	Medium
AUC_0–12_	12.8	16.9	0.13	0.56	Large
AUC_0–24_	29.2	43.8	0.30	0.39	Medium
AUC_0–72_	79.1	116.1	0.58	0.22	Small
AUC_0–96_	94.4	137.7	0.69	0.17	Small
AUC_last_	124.8	159.0	0.94	0.06	Negligible
AUC_inf_	126.6	163.0	0.94	0.06	Negligible
MRT_inf_	62.3	47.0	0.09	−0.61	Large

Abbreviations: Cliff's *δ*, Cliff's delta effect size, Magnitude, magnitude scale level of the effect size for each pharmacokinetic variable; Median, median value for each gender.

## 4 Discussion

This is the first report to describe the pharmacokinetic profile of Ethiqa XR in dogs and to explore sex differences.

The results of this study showed that the therapeutic plasma level of buprenorphine appeared to be sustained for approximately 60–90 h after Ethiqa XR injection at this dose in both males and females, depending on the therapeutic threshold applied. Although no significant differences in buprenorphine plasma concentration were detected between males and females, drug exposure and elimination were greater in females than in males. In both females and males, buprenorphine was rapidly absorbed and distributed into the bloodstream within 5 min of administration, and all dogs maintained detectable plasma concentrations for 168 h (7 days) after administration.

Overall, a greater drug exposure was observed in females (AUCinf: 163.0 h*ng/mL) compared to males (AUCinf: 126.6 h*ng/mL) in this study (Table 4). A similar finding has been described in humans, and Moody et al. (2011) reported the differences in body composition (higher body fat in females), enzyme‐dependent metabolism (P450 (CYP) 3A dependent metabolism), and blood flow to the liver may play a key role in gender‐related pharmacokinetic differences of buprenorphine. To date, no study has reported on sex differences in the pharmacokinetics of buprenorphine in dogs. In the current study, the elimination of buprenorphine was faster in females with a shorter terminal half‐life (24.7 h) than in males (36.6 h). Further studies are warranted to determine the reason.

In Barletta's study (2018), sustained plasma concentrations of buprenorphine (> 2 ng/mL) were noted up to 48 h after a SC injection of an extended‐release buprenorphine (ER‐buprenorphine, Animalgesic Laboratories, Millersville, MD, USA). In contrast, Nunamaker et al. (2014) reported that around the 48 h timepoint, the plasma concentration was approximately 1 ng/mL after a sustained‐release buprenorphine (Buprenorphine SR 3 mg/mL, ZooPharm, Laramie, WY, USA) SC injection. In the present study, the mean plasma concentration at the same post‐dosing time was 1.4 ng/mL in females and 1.8 ng/mL in males. Estimated therapeutic plasma concentrations of buprenorphine remain a controversial topic. Ko et al. (2011) suggested that plasma concentrations > 0.6 ng/mL are associated with analgesic effects in dogs undergoing an ovariohysterectomy. This was based on observations of the Dynamic Interactive Visual Analog Scale assessment system. In another study of dogs undergoing ovariohysterectomy, Nunamaker et al. (2014) reported that 2 out of 20 dogs showed signs of pain at plasma buprenorphine concentrations of 2.08–2.26 ng/mL; in this study, the modified Colorado State University Canine Acute Pain Scale was used to assess pain. Conversely, some dogs did not demonstrate obvious signs of pain even though buprenorphine concentrations were < 0.1 ng/mL (Nunamaker et al. 2014). Barletta et al. (2018) suggested plasma concentrations need to be greater (> 1 ng/mL) after SC administration compared to intravenous administration to see the anti‐nociceptive effects. The authors postulated that this might be related to differences in drug diffusion. Buprenorphine can quickly move to the central nervous system due to the large blood‐nervous system diffusion gradient after intravenous administration compared to SC administration (Barletta et al. 2018).

The situation is complicated by the fact that sensitivity to pain is likely to be variable in dogs and because plasma buprenorphine concentrations do not necessarily reflect pharmacodynamic effects (analgesia or anti‐nociception) (Nunamaker et al. 2014; Hansford et al. 2021; Andaluz et al. 2009). Additionally, anti‐nociception (e.g., hot thermal anti‐nociception) as tested in experimental studies is not the same as clinical pain (Amon et al. 2021), which is influenced by factors such as tissue injury, inflammation, and central sensation.

A significant time lag between maximum concentrations in plasma and maximum drug effect (hysteresis effect) has been reported. Moreover, the duration of action might be longer than that predicted from buprenorphine plasma concentrations due to buprenorphine's high affinity for mu‐receptors and its slow dissociation (Andaluz et al. 2009). Therefore, clinical efficacy may be difficult to predict solely from therapeutic buprenorphine plasma levels and further study is warranted to confirm if the obtained buprenorphine plasma concentrations correlate with clinical antinociception. However, if the therapeutic threshold is set at the plasma concentrations of 0.6 or 1 ng/mL based on previous studies, the efficacy of Ethiqa XR may last up to 60–96 h in males and 72–84 h in females, respectively.

Evaluation of SC administration of Ethiqa XR has been performed in other species. In adult common marmosets ( *Callithrix jacchus* ), Ethiqa XR was administered at either 0.1, 0.15, or 0.2 mg/kg once SC at the ventral abdomen. The *C*_max_ (from 1.4 to 2.5 ng/mL) and AUC_0–72_ (from 47.0 to 86.5 h*ng/mL) of buprenorphine were increased in a dose‐dependent manner, and *C*_max_ was observed between approximately 6–13 h regardless of dose. Buprenorphine concentrations remained above 0.1 ng/mL (assumed therapeutic level in this species) for 72 h after administration for all 3 doses of Ethiqa XR (Fabian et al. 2022).

In rats, reported side effects of Ethiqa XR include mild sedation, bradycardia, hyperthermia, and inappetence (https://ethiqaxr.com/; Collins et al. 2024). Additionally, subcutaneous injection site reactions such as nodules or granulomatous inflammation following administrations have been documented in rats (Levinson et al. 2021; Alamaw et al. 2022). In contrast, no injection site reaction was described in dogs (Barletta et al. 2018) or cynomolgus monkeys (Klein et al. 2023), suggesting this could be dose‐ and species‐dependent variation.

Two long‐lasting buprenorphine formulations are available for cats. Simbadol (1.8 mg/mL, Zoetis, Parsippany, New Jersey, USA) is a concentrated buprenorphine solution approved by the FDA and the medication package insert indicates Simbadol once daily SC injection at 0.24 mg/kg provides up to 24 h of post‐operative pain control in cats and a total of 3 injections can be administered. In beagle dogs, Simbadol (0.12 mg/kg once SC) appeared to provide prolonged plasma concentrations (> 1 ng/mL for approximately 24 h) and longer elimination HL (16 h) (Hansford et al. 2021). Steagall et al. (2020) evaluated the pharmacokinetic profile and analgesic effects of Simbadol administered by SC, IM, or intravenous (IV) routes (0.02 mg/kg) with carprofen (4.4 mg/kg SC once daily for 2 days) in dogs undergoing ovariohysterectomy. The authors concluded that the route of administration influenced the analgesic efficacy of buprenorphine (Simbadol) with SC administration failing to provide clinical analgesia due to erratic drug absorption. They concluded that at the doses of Simbadol administered, the IV and IM routes are preferred for postoperative analgesia (Steagall et al. 2020). However, using Simbadol would require daily IM or IV injections, which is not practical when dogs have left the clinic. The buprenorphine concentrations (mean ± SD (range)) in the dogs that received rescue analgesia (morphine 0.25 mg/kg IV) based on pain assessment (≥ 5/20 the short form of the Glasgow Composite Pain Scale scores) were 0.39 ± 0.17 ng/mL (0.2–0.72 ng/mL) (Steagall et al. 2020). Another long‐acting formulation approved in cats is Zorbium (Elanco, Greenfield, IN), which is a transdermal formulation for postoperative pain for up to 96 h. However, pharmacokinetic or pharmacodynamic data for this product in dogs have not been reported.

In humans, IV buprenorphine administration has been reported to cause small but consistent reductions in HR and arterial pressure (Scott et al. 1980). Similarly, in anesthetized dogs, buprenorphine decreased HR, systolic and diastolic blood pressure, and stroke volume (Martinez et al. 1997). In another canine study, plasma buprenorphine concentration showed poor correlation with RR and HR and only low correlation with SSC following intravenous or SC injections; however, some individuals exhibited mild bradycardia and hypothermia (Barletta et al. 2018). Similar to these studies, in the current study, within individuals, decreased body temperature and lower HR were associated with higher buprenorphine concentrations, without corresponding changes in RR. These changes we found are mild, and we do not consider them clinically relevant in healthy dogs (Martinez et al. 1997). Buprenorphine has been shown to result in sedation, with generally higher sedation scores observed in the first 3–4 h after administration (Ko et al. 2011; Nunamaker et al. 2014), which is similar to our study, where we saw the highest sedation score at 4–6 h post‐administration. In humans, the onset of pharmacodynamic effects (including sedation) of buprenorphine can occur during the distribution phase, while plasma concentration is still rising, indicating a temporal dissociation consistent with counterclockwise hysteresis (Escher et al. 2007).

Opioids are known to induce nausea and vomiting through stimulation of the chemoreceptor trigger zones in the area postrema of the medulla. (Watcha and White 1992) In our study, higher plasma buprenorphine concentrations were positively correlated with NSC (*r* = 0.237) and SSC (*r* = 0.513) scores in the female dogs, while no relationship was present in male dogs. Interestingly, in humans, studies have reported a higher frequency of nausea and vomiting in females receiving post‐operative opioids (Koivuranta et al. 1997; Zun et al. 2002; Lopes et al. 2021; Romanescu et al. 2022). Additionally, post‐marketing data regarding buprenorphine indicate that women reported nausea and vomiting 10%–15% more frequently than males (https://www.drugs.com/sfx/buprenex‐side‐effects.html; Thornton 2025). While the mechanisms remain unclear, preclinical studies demonstrate that sex hormones such as estrogen can modulate μ‐opioid receptor signaling (Acosta‐Martinez and Etgen 2002), which may explain sex differences in adverse effects. Some studies in humans and rhesus monkeys have reported that the sensitivity to mu‐opioid receptors can be changed depending on gender (female is more sensitive), age (older is more sensitive), and ovarian hormone (progesterone, androgens, estrogens) status (mu‐receptor binding decreases after menopause) (Ethridge and Smith 2023; Zubieta et al. 1999; Schwienteck et al. 2019). However, all the scoring was performed by a single, non‐blind person, using a subjective scale which might induce bias and create a coincidence between buprenorphine concentration and scores. Additionally, the scoring systems that we used have not been fully validated and, thus, may not be reliable. The results would be strengthened if multiple people were involved or if objective methods were used. However, to the best of our knowledge, there are no objective methods to assess a dog's sedation and nausea levels yet.

To our knowledge, no study has directly compared the incidence of vomiting and nausea between sexes in dogs post buprenorphine administration. Further research can be considered to confirm if co‐administration of maropitant with Ethiqa XR could reduce the occurrence of gastrointestinal clinical signs and manifestations. In rats, urine output suppression is reported, but ureteric peristalsis was not observed in previous studies (Sramek et al. 2015). The cause of apparent temporary straining in one female (female 5) was unclear and may have been related to the observed tenesmus.

As reported for other formulations of buprenorphine administered in dogs and cats, gastrointestinal issues, including vomiting, inappetence, and diarrhea or soft feces were observed in the current study (Enomoto et al. 2022; Barletta et al. 2018; Nunamaker et al. 2014). In the study by Nunamaker et al., soft stool or diarrhea occurred in 17 of 20 dogs. Other studies reported gastrointestinal adverse events but did not specify incidence (Enomoto et al. 2022, and Barletta et al. 2018). In addition to soft feces and diarrhea in our dogs, straining to defecate was observed, which has not been previously reported in canines, however, it should have been reported in a published efficacy study, as review of the data (unpublished files) described straining to defecate/tenesmus in some dogs (Tomas et al. 2015). Straining to defecate was described in humans as one of the clinical symptoms of opioid‐induced bowel dysfunction (Pappagallo 2001).

There are limitations associated with the present study. Our study had a small sample size, typical of PK studies in healthy research dogs, which likely explains the lack of statistical significance in comparing PK parameters between males and females. Only a single non‐blind person was involved in scoring and assessments; the results would be strengthened if multiple people performed them. Because our dogs were young, healthy beagles, we cannot be certain the results are fully generalizable to the population of dogs this product may be used in; however, such a product, if available, may be used most extensively in young dogs undergoing desexing. Our dogs were not suffering any pain, and so further evaluation of side effects in dogs being treated for a pain condition should be performed as the severity and frequency of adverse events and the PK may be different. Additionally, further studies are needed to determine the efficacy of Ethiqa XR in dogs.

Although there is more work to be done to define the efficacy and optimal dose of Ethiqa XR in dogs, such treatment, if effective, may provide a much‐needed tool to address the current gap in our ability to provide opioid‐based pain relief following surgery in canine outpatients. Thus, Ethiqa XR would improve animal welfare.

## 5 Conclusion

This study demonstrated Ethiqa XR can be administered to healthy dogs. Estimated therapeutic plasma concentrations were maintained for at least 60 h (60–96) in males and at least 48 h (48–96) in females following a 0.2 mg/kg dose administered SC. Further work is warranted to assess the analgesic efficacy of Ethiqa XR in dogs, and to further define side effects in a population being treated for pain condition.

## Author Contributions

K.T. performed clinical examinations, monitored side effects using multiple scoring during the animal study, conducted data collection and analysis, and wrote the manuscript. H.E. conducted the pharmacokinetic sampling, developed the buprenorphine analytical method and analyzed samples, performed P.K. analysis, and participated in drafting and editing the manuscript. L.M.N. assisted with preparing the dogs for sampling and reviewed the manuscript. R.E.B. assisted with study design and reviewed the manuscript. B.D.X.L. and M.E. developed the experimental concept and study design, oversaw all aspects of the project, and guided and edited the manuscript.

## Funding

This work was supported by Fidelis Animal Health, Inc. (PAM‐P25‐002754).

## Ethics Statement

The study was approved by IACUC at North Carolina State University, protocol 25‐231.

## Conflicts of Interest

The authors declare no conflicts of interest.

## Supporting information


**File S1:** Seven‐point sedation scale validated in dogs by Wagner et al. 2017.
**File S2:** Inter‐day precision and accuracy of analytical method for buprenorphine canine plasma samples.
**Figure S1:** (a–d) Individual buprenorphine plasma concentration (ng/mL) versus time (hours) curve after a single subcutaneous administration of extended‐release buprenorphine (Ethiqa XR; 0.2 mg/kg) in intact male dogs and intact female dogs. The dashed line represents the limit of quantification (LOQ, 0.1 ng/mL) of the assay. Therapeutic level: the plasma concentration (0.6 ng/mL, dotted) that has been reported to be associated with analgesic effects in dogs for ovariohysterectomy (Ko et al. 2011). (a) intact male dogs for the entire study period. (b) intact female dogs for the entire study period. (c) up to 8 h for male dogs. (d) up to 8 h for female dogs. All data above the limit of detection (LOD, 0.01 ng/mL) were used for this figure.

## Data Availability

The findings of this study are available from the corresponding author upon reasonable request.
