# Supplementary material for: Pharmacokinetics of a Single Subcutaneous Extended‐Release Buprenorphine (Ethiqa XR) in Healthy Beagles
Source: J Vet Pharmacol Ther. 2026 May 20;49(5):471–86. doi: 10.1111/jvp.70082 (PMC13552516; doi:10.1111/jvp.70082)
Supplement: Supplementary file 1 [file JVP-49-471-s001.docx]

**File S1: Seven-point sedation scale validated in dogs by Wagner et al., 2017**

| **1. Spontaneous Posture** | Standing = 0  Tired but standing = 1  Lying but able to rise = 2  Lying but difficult rising = 3  Unable to rise = 4 |
| --- | --- |
| **2. Palpebral Reflex** | Brisk = 0  Slow but with full corneal sweep =1  Slow but with only partial corneal sweep =2  Absent =3 |
| **3. Eye Position** | Central =0  Rotated forwards/downwards but not obscured by third eyelid =1  Rotated forwards/downwards and obscured by third eyelid =2 |
| **4. Jaw and tongue relaxation** | Normal jaw tone, strong gag reflex = 0  Reduced tone, but still moderate gag reflex =1  Much reduced tone, slight gag reflex =2  Loss of jaw tone and no gag reflex = 3 |
| **5. Response to noise (handclap)** | Normal startle reaction (head turn towards noise/ cringe) = 0  Reduced startle reaction (reduced head turn/ minimal cringe) = 1  Minimal startle reaction =2  Absent reaction =3 |
| **6. Resistance when laid into lateral recumbency*** | Much struggling, perhaps not allowing this position =0  Some struggling, but allowing this position =1  Minimal struggling/ permissive =2  No struggling = 3 |
| **7. General appearance / attitude** | Excitable =0  Awake and normal =1  Tranquil =2  Stuporous =3 |

***note:** However, this was not applicable in these beagles, as they were trained for this procedure, and showed no resistance at any time during study. Therefore ‘resistence to being placed in lateral recumbancy’ was removed from the scale, and our maximum sedation score was 18.

**File S2**. Inter-day precision and accuracy of analytical method for buprenorphine canine plasma samples

| Nominal concentration of buprenorphine (ng/mL) | Average observed concentration of buprenorphine (ng/mL) | Average recovery (%) | Coefficient of variation (%) |
| --- | --- | --- | --- |
| 0.05 | 0.043 | 86.08 | 15.64 |
| 0.1 | 0.093 | 93.68 | 7.39 |
| 0.3 | 0.324 | 107.86 | 6.20 |
| 0.25 | 0.262 | 104.78 | 7.47 |
| 0.5 | 0.514 | 102.88 | 3.63 |
| 1 | 1.004 | 100.42 | 3.39 |
| 3 | 3.679 | 122.64 | 11.02 |
| 5 | 5.372 | 107.44 | 3.50 |
| 7 | 7.878 | 112.55 | 10.32 |
| 10 | 10.69 | 106.88 | 3.00 |
| 30 | 33.56 | 111.86 | 11.58 |
| 50 | 48.92 | 97.84 | 0.87 |

**Figure S1. (a–d) Individual buprenorphine plasma concentration (ng/ml) versus time (hours) curve after a single subcutaneous administration of extended-release buprenorphine (Ethiqa XR^®^; 0.2 mg/kg) in intact male dogs and intact female dogs. The dashed line represents the limit of quantification (LOQ, 0.1 ng/ml) of the assay. Therapeutic level: the plasma concentration (0.6 ng/ml, dotted) that has been reported to be associated with analgesic effects in dogs for ovariohysterectomy (Ko et al., 2011). a: intact male dogs for entire study period. b: intact female dogs for entire study period. c: up to 8 hours for male dogs. d: up to 8 hours for female dogs. All data above limit of detection (LOD, 0.01 ng/mL) were used for this figure.**
